# 692. The Role of Fidaxomicin for the Treatment of Clostridioides Difficile Infection in the Pediatric Population in the United States

**DOI:** 10.1093/ofid/ofad500.754

**Published:** 2023-11-27

**Authors:** Yuman Lee, Nicole Bradley, Sarah Smith, Shakhzoda Rakhimova

**Affiliations:** St. John's University, Queens, New York; St. John's University, Queens, New York; St. John's University, Queens, New York; St. John's University, Queens, New York

## Abstract

**Background:**

In 2020, the FDA approved fidaxomicin (FDX) for the treatment of Clostridioides difficile infection (CDI) in children 6 months and older. The latest IDSA/SHEA guidelines recommend FDX use for adults only. A survey presented at IDWeek 2022 showed that only 35% of institutions used FDX as first line in adults despite its inclusion in the guidelines. The purpose of this study is to assess the use of FDX in US pediatric patients and identify barriers to use.

**Methods:**

An anonymous, voluntary electronic survey was distributed to the American Society of Health-System Pharmacists and the Pediatric Pharmacy Association in January 2023. Baseline demographics, current practices, knowledge, comfort, and barriers to FDX use in pediatrics were collected. Fisher’s Exact Test was used to compare categorical variables.

**Results:**

32 pharmacists responded to the survey. Majority were from children’s hospitals within an adult hospital (62.5%), stand-alone children's hospitals (18.8%), and adult hospitals with pediatric beds (15.6%). 37.5% (12/32) reported having institutional pediatric CDI guidelines. Of which, 75% (9/12) included FDX for the treatment of previous failure 77.8% (7/9), recurrent CDI 44.4% (4/9), and fulminant CDI 22.2% (2/9). In institutions without pediatric CDI guidelines (62.5%, 20/32), FDX was used for the treatment of previous failure 65% (13/20), recurrent CDI 5% (1/20), and fulminant CDI 5% (1/20). FDX use was significantly higher in institutions with CDI guidelines for recurrent CDI compared to those without guidelines (p< 0.05).

96.9% (31/32) of respondents reported at least one barrier to FDX use. Top 3 barriers were cost (71%, 22/31), inability of patients to afford upon discharge (64.5%, 20/31), and lack of provider familiarity (41.9%, 13/31). 53.1% (17/32) respondents were comfortable using FDX in pediatrics, 87.5% (28/32) were aware of its FDA approval in children, and 90.6% (29/32) were aware it was not in the IDSA guidelines.

**Conclusion:**

Most respondents did not have institutional pediatric CDI guidelines. FDX use was most common in treatment failure. Most respondents reported at least one barrier to FDX use despite its FDA approval. Majority were comfortable using FDX in pediatrics. This study is limited by its small sample size as expected due to the studied population.

**Disclosures:**

**All Authors**: No reported disclosures

